# The effect of antibiotic therapy on clinical outcome in patients hospitalized with moderate COVID-19 disease: a prospective multi-center cohort study

**DOI:** 10.1007/s15010-025-02590-0

**Published:** 2025-06-26

**Authors:** Anette Friedrichs, Roman Wenz, Daniel Pape, Katharina S. Appel, Thomas Bahmer, Karsten Becker, Sven Bercker, Sabine Blaschke, Josephine Braunsteiner, Jana Butzmann, Egdar Dahl, Johanna Erber, Lisa Fricke, Ramsia Geisler, Siri Göpel, Andreas Güldner, Marina Hagen, Axel Hamprecht, Stefan Hansch, Peter U. Heuschmann, Sina M. Pütz, Björn-Erik Ole Jensen, Nadja Käding, Julia Koepsell, Carolin E. M. Koll, Marcin Krawczyk, Thomas Lücke, Patrick Meybohm, Milena Milovanovic, Lazar Mitrov, Carolin Nürnberger, Christoph Römmele, Margarete Scherer, Lena Schmidbauer, Melanie Stecher, Phil-Robin Tepasse, Andreas Teufel, Jörg Janne Vehreschild, Christof Winter, Oliver Witzke, Christoph Wyen, Frank Hanses, Amke Caliebe

**Affiliations:** 1 Department of Internal Medicine, University Hospital Schleswig-Holstein, Campus Kiel, Kiel, Germany; 2 Institute of Medical Informatics and Statisitics, University Hospital Schleswig-Holstein, Campus Kiel, Kiel, Germany; 3 Institute for Digital Medicine and Clinical Data Science, University Hospital Frankfurt, Goethe University, Frankfurt am Main, Germany; 4 Airway Research Center North (ARCN), German Center for Lung Research (DZL), Grosshansdorf, Germany; 5 Friedrich Loeffler-Institute of Medical Microbiology, University Medicine Greifswald, Greifswald, Germany; 6 University Hospital Leipzig, Leipzig, Germany; 7 Emergency Department, University Medical Center Goettingen, Göttingen, Germany; 8 Department of Intensive Care Medicine, University Medical Center Hamburg-Eppendorf, Hamburg, Germany; 9 Institute of Medical Microbiology and Hospital Hygiene, University Hospital Magdeburg, Magdeburg, Germany; 10 Institute of Pathology, University Hospital Aachen, Aachen, Germany; 11 TUM School of Medicine, Clinical Department for Internal Medicine II, University Hospital rechts der Isar, Technical University of Munich, Munich, Germany; 12 Division of Infectious Diseases, Department of Internal Medicine I, University Hospital Tübingen, Tübingen, Germany; 13 Department of Anesthesiology and Intensive Care Medicine, University Hospital “Carl Gustav Carus”, Technische Universität Dresden, Dresden, Germany; 14 Department II of Internal Medicine, Hematology/Oncology, Goethe University, Frankfurt am Main, Germany; 15 Institute for Medical Microbiology and Virology, University Medicine Oldenburg, Oldenburg, Germany; 16 Department for Infectious Diseases and Infection Control, University Hospital Regensburg, Regensburg, Germany; 17 Institute for Clinical Epidemiology and Biometry, University of Wurzburg, Würzburg, Germany; 18 Department I for Internal Medicine, Faculty of Medicine and University Hospital of Cologne, University of Cologne, Cologne, Germany; 19 Department of Gastroenterology, Hepatology and Infectious Diseases, Düsseldorf University Hospital, Düsseldorf, Germany; 20 Infectious Diseases Clinic, University of Schleswig -Holstein, Campus Lübeck, Lübeck, Germany; 21 Department of Medicine II, Saarland University Medical Center, Saarland University, Homburg, Germany; 22 Department of Pediatrics, Ruhr University, Bochum, Germany; 23 Department of Anaesthesiology, Intensive Care, Emergency and Pain Medicine, University Hospital Würzburg, Würzburg, Germany; 24 Departement I of Internal Medicine, Malteser Clinic St. Franziskus Hospital, Flensburg, Germany; 25 Gastroenterology and Infectious diseases, Faculty of Medicine, University Hospital of Augsburg, Augsburg, Germany; 26 Department of Medicine B for Gastroenterology, Hepatology, Endocrinology and Clinical Infectiology, University Hospital Münster, Münster, Germany; 27 Division of Hepatology, Department of Medicine II, Medical Faculty Mannheim, Heidelberg University, Mannheim, Germany; 28 TUM School of Medicine, Institute for Clinical Chemistry and Pathobiochemistry, University Hospital rechts der Isar, Technical University of Munich, Munich, Germany; 29 Department of Infectious Diseases, West German Centre of Infectious Diseases, University Medicine Essen University Hospital Essen, University Duisburg-Essen, Essen, Germany; 30 Praxis Ebertplatz, Cologne, Germany

**Keywords:** COVID-19, Rational antibiotic therapy, Clinical outcome, Moderate disease, Clinical improvement

## Abstract

**Purpose:**

The benefit of antibiotic treatment (ABT) for patients with moderate COVID-19 is unclear and overtreatment poses the risk of adverse effects such as *Clostridioides difficile* infection and antibiotic resistance. This multi-center study compares health status improvement between patients with and without ABT at hospital admission.

**Methods:**

Between March 2020 and May 2023, hospitalized adults with confirmed SARS-CoV-2 infection were recruited from the German National Pandemic Cohort Network (NAPKON), which includes patients from various hospitals across Germany. The study population included patients with moderate or severe COVID-19 at baseline. The primary objective was to compare health improvement or decline after two weeks between patients who received ABT at baseline and those who did not in the moderate COVID-19 population. The statistical analysis adjusted for confounders such as gender, age, vaccination status, clinical condition, and comorbidities. The severe COVID-19 population was investigated as a secondary objective.

**Results:**

A total of 1,317 patients (median age 59 years; 38% women) were eligible for analysis, of whom 1,149 had moderate and 168 severe COVID-19 disease. ABT for pneumonia was administered to 467 patients with moderate and 117 with severe COVID-19. ABT at baseline was significantly associated with a higher deterioration rate after two weeks in patients with moderate COVID-19 (ABT: 292 improvement, 61 deterioration; no ABT: 429 improvement, 14 deterioration). A similar result was obtained in the multiple regression analysis where an odds ratio of 5.00 (95% confidence interval: 2.50 – 10.93) for ABT was observed.

**Conclusion:**

We found no benefit of antibiotic therapy in patients with moderate COVID-19. Use of ABT was associated with a higher likelihood of clinical deterioration.

**Graphical abstract:**

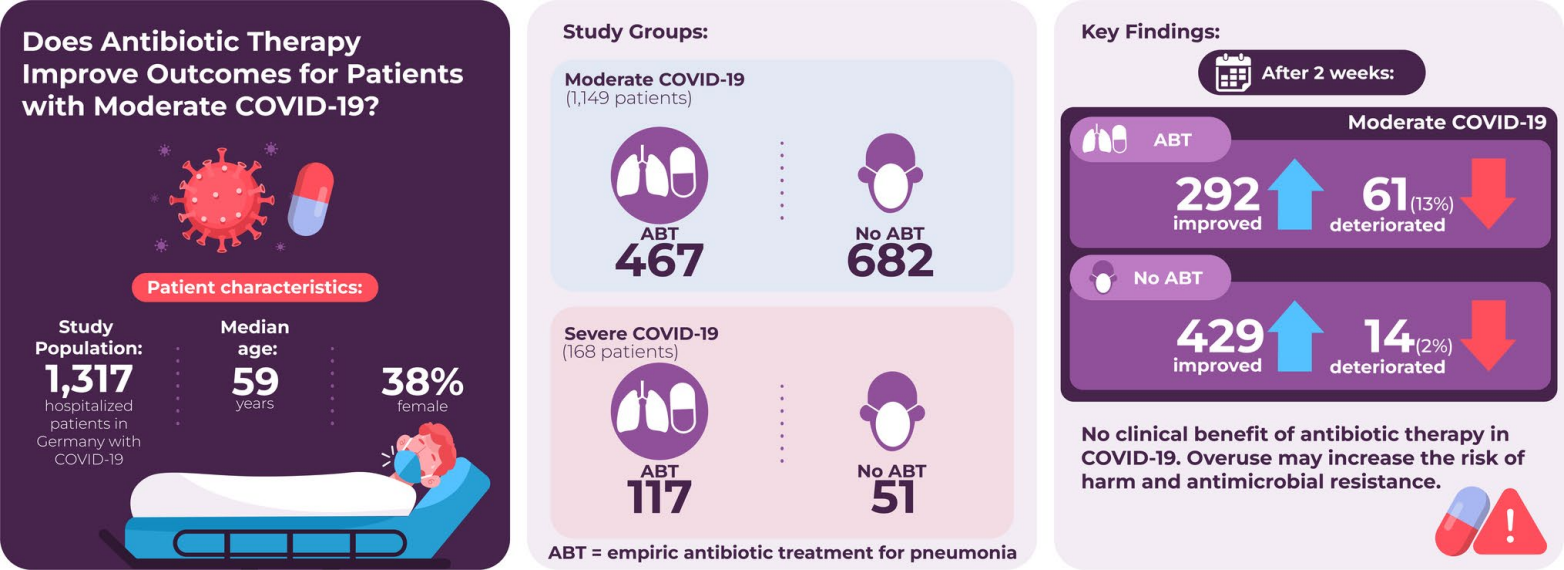

**Supplementary Information:**

The online version contains supplementary material available at 10.1007/s15010-025-02590-0.

## Introduction

Despite COVID-19 being a viral disease, the percentage of hospitalized patients receiving systemic antibiotic therapy (ABT) remains surprisingly high, often exceeding 60% [1–3]. According to German guidelines, preemptive ABT should be avoided and only be prescribed in cases of suspected or confirmed bacterial co- or superinfection in COVID-19 patients [4]. The estimation of co- and superinfection rates (3.1–6.9%) is hampered by frequently incomplete documentation and depends on clinical conditions and treatment [5, 6]. However, clinical symptoms of bacterial superinfections and advanced stages of COVID-19 can be similar leading to a discrepancy between actual superinfection rates and the usage of antibiotics in COVID-19 patients which exacerbates the development of antibiotic resistance.

The WHO Clinical Progression Scale (WHO score) [7] ranges between 0 and 10, reflecting COVID-19 severity and differentiates three levels of clinical intervention required: mild (1–3, ambulatory care), moderate (4, 5, hospitalized and may need oxygen delivered by mask) and severe (6–9, need for non-invasive ventilation, mechanical ventilation or organ support). Death is rated by a score of ten. ABT is typically not required in mild cases of COVID-19. For severe cases, bacterial co- and superinfections are known to have a largely increased mortality risk making it ethically problematic to refrain from initial empiric ABT. Therefore, patients with moderate COVID-19 present the greatest challenge for doctors when determining if early ABT is appropriate. Our study aims to demonstrate whether empiric antibiotic use for suspected bacterial pulmonary superinfection impacts on health decline when used in patients with moderate or severe COVID-19 according to WHO classification. Considering the clinical relevance, the effect in patients with moderate COVID-19 (WHO 4, 5) was the primary objective, analysis of effects in severe COVID-19 (WHO 6–9) was the secondary objective.

## Methods

In this study, we address this critical question using the largest German cohort available through the German National Pandemic Cohort Network (NAPKON) [8]. Patients were grouped based on whether they received ABT upon hospital admission or not. Primary outcome of our study was improvement or decline in health after two weeks as measured by change in WHO score.

### WHO clinical progression scale

The WHO score was used in this study to assess patient severity (Online Resource Supplementary Table S1) [7].

### Study objectives

#### Primary objective

The primary objective of the study was to compare clinical outcomes between patients who did and did not receive antibiotic treatment (ABT) at baseline (i.e., at study enrollment). The study population consisted of patients with moderate COVID-19 at baseline (WHO score 4, 5). The primary outcome was improvement or decline in health defined by an increasing or decreasing change in WHO score between baseline visit and two weeks later.

#### Secondary objectives

The secondary objectives were the following:

- Compare patients with moderate COVID-19 at baseline with and without ABT with respect to the outcomes health decline (decreased WHO score) and death during the observation period (hospital stay and follow-up).
- The same as for primary and first secondary objectives but for the population of patients with severe COVID-19 disease at baseline visit (WHO score 6–9).

### Study cohort

The National Pandemic Cohort Network (NAPKON, https://napkon.de/projekt-napkon), established a comprehensive cross-sectoral COVID-19 cohort in Germany (SUEP), tracking patients from infection onset up to three years. The study employs standardised procedures and biosample storage. This cohort represents a national, multicenter, minimally interventional prospective cohort study of patients diagnosed with SARS-CoV-2 infection, including procedures such as taking blood cultures and swabs, collection of urine and respiratory samples and surveys. The study design allowed for patients to receive a baseline visit and defined follow up visits (weekly, if clinical deterioration, end of acute phase, discharge, death if applicable, telephone follow-ups every six weeks post-infection, at three and 12 months). All patients gave written informed consent prior to their inclusion.

Our study was approved by the Ethics Committee of the Medical Faculty of the University Hospital Schleswig–Holstein, Kiel, Germany (Approval Number: D595/21).

### Inclusion and exclusion criteria

We considered visit data from March 2020 to May 2023. Inclusion in the study required a positive SARS-CoV-2 PCR test at hospital admission, at least a baseline and a discharge visit, being hospitalized, at least 18 years old, and available information on ABT seven days prior to baseline to four days after baseline. Patients had to be classified according to moderate (WHO score 4, 5) or severe (WHO score 6–9) COVID-19. Patients with directed ABT due to foci other than the lung (e.g. with pathogenic organisms in stool or ascites) were excluded. Furthermore, patients on palliative care were excluded due to other aims of therapy. Quality checks of data identified one patient with an implausible baseline date who was excluded from the study.

### Antibiotics and microbial diagnostics

In our study, we focused solely on antibiotics commonly used in the treatment of respiratory infections. The antibiotics included were acylureidopenicillines combined with a β-lactamase inhibitor (BLI), aminopenicillines with or without BLI, carbapenemes, second to third generation cephalosporines (including subgroup 3b), fluoroquinolones, macrolides, and tetracyclines (Online Resource Supplementary Table S2). With regard to relevant pathogens, we took into account microbiological results from seven days before and four days after the baseline visit. Three infectious disease specialists defined relevant diagnostic materials and independently rated whether pathogens were the likely origin of pneumonia and required appropriate ABT. Differences were resolved through discussion.

### Statistical analyses

Assuming a 20% proportion of health decline in the study population of patients with moderate COVID-19 who experienced a change in health status within two weeks, a sample size of at least 219 patients in both the ABT and non-ABT groups is required to detect a 10% difference in the proportion of health decline. This calculation is based on a significance level of 0.05 and a statistical power of 0.80, using a Chi-squared test with Yates correction (BIAS for Windows, Version 11.12).

The WHO score was calculated for each patient at every documented visit. We defined deterioration or improvement by comparing the WHO score at baseline with a later visit. For the primary outcome, time interval was set at 14 days after baseline. If no visit occurred exactly 14 days post-baseline, the visit closest prior to 14 days post-baseline was included in the analysis. Since additional visits were conducted if the patient’s health deteriorated, the visit closest to the 14-day mark is likely to reflect the patient’s health status at that time. For any discharge or death occurring within the 14-day period the WHO score was adjusted accordingly.

All variables were categorised in groups. Descriptive statistics show absolute and relative percentages. For univariable analyses with ABT or clinical improvement/decline as outcome we applied Fisher’s exact test. Multiple logistic regression with ABT status (yes/no) as outcome was performed to identify factors relevant to ABT prescription. Furthermore, multiple logistic regression analysis was used with improvement/decline after two weeks (primary outcome) and health decline and death during hospital stay (secondary outcomes) to investigate the influence of ABT adjusted for additional covariables and potential confounders.

In both cases, we chose clinically relevant factors as influence variables. These had to be sufficiently documented with a sufficient frequency of recorded events. We therefore summarized single comorbidities in the updated Charlson Comorbity Index CCI. No interactions were considered and no imputation for missing values was used. For the logistic model, we applied backward selection with a significance threshold of 0.05. Results of multiple regression analyses are presented as odds ratios with 95% confidence intervals. Additionally, Nagelkerke’s R^2^ was calculated as a measure of goodness of fit. The outcome death was additionally analysed by the Kaplan–Meier method and groups with and without ABT were compared with a log rank test. No Cox regression was performed because the proportional hazard assumption was strongly violated.

To test the robustness of our results and especially to account for differences in risk factors like comorbidities between patients receiving ABT and those who did not, a propensity score analysis with matching was conducted. For this, the R package MatchIt was utilized [9]. The propensity score was calculated using a logistic regression model and all covariables significant in the univariable analysis (Table 2; age, gender, documented pathogen detection, vaccination, frailty, CCI). For matching, a ratio of 1:1 with respect to patients with and without ABT was applied and we utilized the nearest neighbor approach and a caliper of 0.1. The matched dataset included 426 patients (213 with and 213 without ABT). After matching, the groups of patients with and without ABT showed good balance with respect to the selected covariables (Online Resource Supplementary Table S3). The primary outcome improvement vs. decline in health after two weeks was then compared between patients with and without ABT on the matched data by first performing a logistic regression with improvement/decline as outcome and ABT and covariables as influence variables, utilizing the weights of the propensity score matching and applying the argument ‘family = quasibinomial’ for more robustness. After that group comparison was performed with the function avg_comparison of the R package marginaleffects [10] to derive odds ratios and the 95% confidence interval using the subclasses of the propensity matching for the argument ‘vcov’. This accounts for matched individuals by a cluster-robust method. We repeated the analysis excluding patients with documented pathogens.

All analyses used a two-sided significance level of p = 0.05 and were performed with the statistics software R, Version 2023.06.1 + 524 [11].

## Results

### Characteristics of participants

**Fig. 1:**
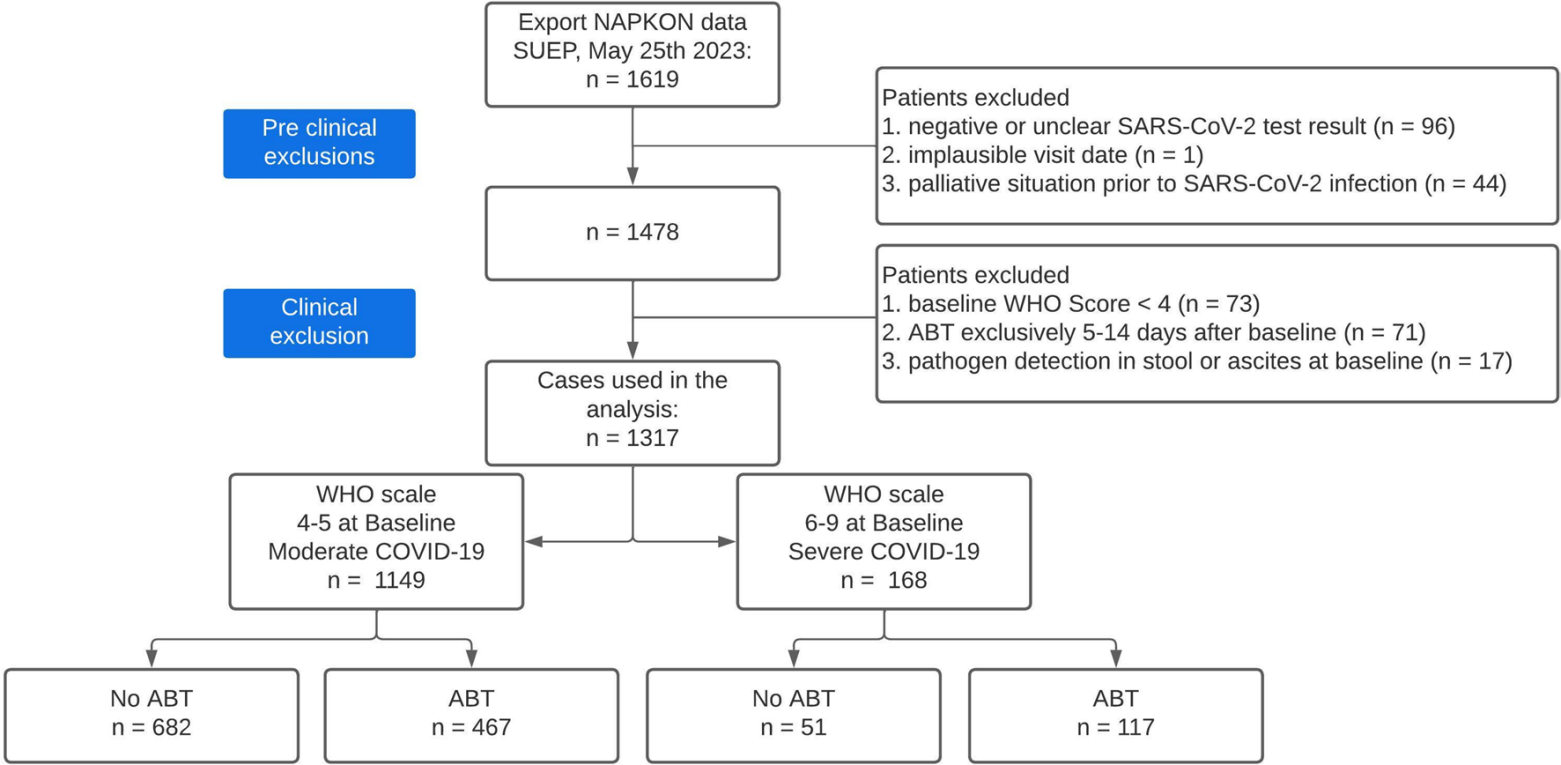
Flowchart of study participants. *ABT* antibiotic treatment, *NAPKON* National Pandemic Cohort Network, *SUEP* Cross-sectoral cohort, *WHO* World Health Organization

Initially, 1619 patients were screened for suitability. Pre-clinical and clinical exclusion criteria and distribution to study cohorts are shown in the flow chart (Fig. 1). COVID-19 severity was assessed by WHO score (Online Resource Supplementary Table S1). Only patients with moderate or severe COVID-19 (WHO score 4–9) were enrolled, excluding 73 additional patients. Finally, 1317 patients were eligible for analysis with 1149 having moderate COVID-19 (WHO score 4, 5) at baseline and 168 presenting with severe COVID-19 (WHO score 6–9). Details on demographic and clinical information are documented in Table 1. Altogether, 584 patients received ABT, including 467 with moderate and 117 with severe COVID-19 (Online Resource Supplementary Table S2); 45 pneumonia-relevant pathogens were identified in relevant samples (Online Resource Supplementary Table S4).

**Table 1:** Characteristics of patients at baseline

	Moderate COVID-19 n = 1,149		P-value	Severe COVID-19 n = 168		P-value
	No ABT n = 682	ABT n = 467		No ABT n = 51	ABT n = 117	
Gender			0.001			0.5
Female	295 (65%)	158 (35%)		17 (35%)	32 (65%)	
Male	387 (56%)	309 (44%)		34 (29%)	85 (71%)	
Age group			< 0.001			0.047
18–49.9	279 (73%)	105 (27%)		19 (43%)	25 (57%)	
50–64.9	222 (58%)	158 (42%)		22 (33%)	45 (67%)	
65–79.9	134 (50%)	135 (50%)		8 (19%)	35 (81%)	
⩾80	47 (41%)	69 (59%)		2 (14%)	12 (86%)	
Respiratory pathogen documented			0.059			< 0.001
Yes	3 (27%)	8 (73%)		0 (0%)	23	
No	679 (60%)	459 (40%)		51 (35%)	94 (65%)	
BMI			0.6			0.6
< 20	27 (52%)	25 (48%)		1 (50%)	1 (50%)	
20–29.9	348 (60%)	235 (40%)		18 (28%)	47 (72%)	
30–34.9	94 (56%)	73 (44%)		14 (37%)	24 (63%)	
> 35	71 (61%)	45 (39%)		10 (37%)	17 (63%)	
Unknown	142	89		8	28	
Vaccination			0.020			0.6
No	175 (55%)	143 (45%)		17 (32%)	36 (68%)	
Yes	387 (63%)	227 (37%)		24 (38%)	39 (62%)	
Unknown	120	97		10	42	
Smoking			0.089			0.9
No, never	323 (63%)	186 (37%)		20 (38%)	32 (62%)	
No, former	169 (56%)	135 (44%)		13 (34%)	25 (66%)	
Yes, active	59 (61%)	38 (39%)		5 (42%)	7 (58%)	
Unknown	131	108		13	53	
Clinical Frailty Scale			< 0.001			0.027
Uncomplicated	501 (63%)	297 (37%)		26 (47%)	29 (53%)	
Complicated	66 (48%)	71 (52%)		8 (24%)	25 (76%)	
Critical	15 (31%)	34 (69%)		9 (23%)	30 (77%)	
Unknown	100	65		8	33	
CCI			< 0.001			0.003
0–2	460 (65%)	253 (35%)		42 (39%)	65 (61%)	
3–4	138 (51%)	135 (49%)		7 (17%)	34 (83%)	
> 4	84 (52%)	79 (48%)		2 (10%)	18 (90%)	
Baseline WHO score			0.001			< 0.001
4	402 (64%)	230 (36%)		–	–	
5	280 (54%)	237 (46%)		–	–	
6–7	–	–		46 (38%)	75 (62%)	
8–9	–	–		5 (11%)	42 (89%)	

*CCI* charlson comorbidity index, *WHO* world health organization, *BMI* body mass index, *ABT* antibiotic treatment, *P-value* P-value for group differences between patients with and without ABT (Fisher exact test)

**Fig. 2:**
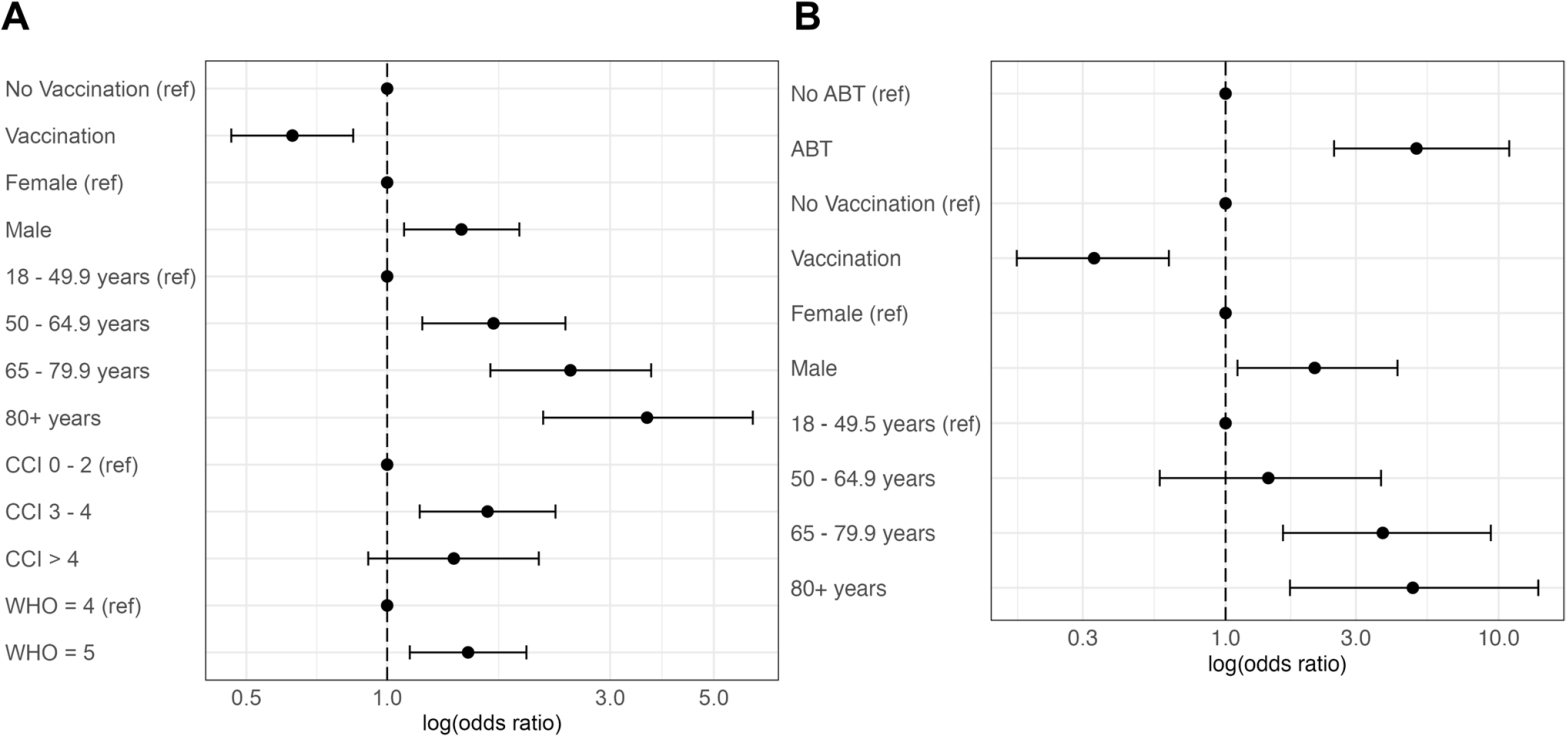
Forest plots for moderate COVID-19 at baseline. Results from multiple logistic regressions. Shown are logarithmic (base 10) odds ratios and 95% confidence intervals of the final multiple model after model selection. **A** Factors associated with ABT at baseline; variables in the full model but excluded during model selection: body mass index, smoking, Clinical Frailty Scale. **B** Factors associated with improvement vs. deterioration after 14 days; variables in the full model but excluded during model selection: Charlson Comorbidity Index, body mass index, smoking, Clinical Frailty Scale, baseline WHO (World Health Organisation) score. Results from a multiple logistic regression. ref: Reference category for the variable in the multiple logistic regression model, CCI: Charlson comorbidity index, WHO: baseline WHO (World Health Organization) score

When comparing patients receiving ABT to those who did not, male gender, higher age, no SARS-CoV-2 vaccination, higher frailty score, more comorbidities and a higher baseline WHO score showed a significant nominal univariable association with ABT in the group of patients with moderate COVID-19 (Table 1). In the multiple logistic regression analysis, frailty was no longer significant (Fig. 2A, Online Resource Supplementary Table S5). In patients with severe COVID-19, significant influential factors for ABT were similar in the univariable analysis (higher age, documented pathogen detection, higher frailty, more comorbidities and higher baseline WHO score), whereas in the multiple analysis only the CCI and the baseline WHO score remained in the final model after backward selection (Online Resource Supplementary Table S6).

### Improvement or decline of health after two weeks from hospital admission

Of 1149 patients with moderate COVID-19, 796 improved and 75 worsened in health after two weeks based on WHO score. Comparing improved and deteriorated patients, male gender, higher age, documented pathogen detection, no vaccination, higher frailty and more comorbidities were significantly more frequent in patients with worsened health (Table 2). ABT was also more frequent in these patients. Because the latter might be due to confounding effects of health status (either due to COVID-19 or overall) or demographic factors, we performed a multiple logistic regression. Here, gender, age, vaccination and antibiotics remained in the final model. Higher age was a risk factor with odds ratios (ORs) between 1.43 and 4.85 for higher age categories as well as male gender (OR 2.12). Instead, vaccination was a protective factor (OR 0.33). ABT showed an OR of 5.00 indicating that patients with ABT had five-fold higher odds for worsening (Online Resource Supplementary Table S7, Fig. 2B).

**Table 2:** Clinical improvement vs. deterioration after 14 days

	Moderate COVID-19 n = 796		P-value	Severe COVID-19 n = 140		P-value
	Improvement n = 721	Deterioration n = 75		Improvement n = 91	Deterioration n = 49	
Gender			0.009			0.083
Female	295 (94%)	19 (6.1%)		32 (76%)	10 (24%)	
Male	426 (88%)	56 (12%)		59 (60%)	39 (40%)	
Age group			< 0.001			0.5
18–49.9	236 (96%)	10 (4.1%)		27 (73%)	10 (27%)	
50–64.9	259 (93%)	21 (7.5%)		35 (63%)	21 (38%)	
65–79.9	168 (86%)	28 (14%)		23 (66%)	12 (34%)	
⩾ 80	58 (78%)	16 (22%)		6 (50%)	6 (50%)	
ABT at baseline			< 0.001			< 0.001
No	429 (97%)	14 (3.2%)		43 (93%)	3 (6.5%)	
Yes	292 (83%)	61 (17%)		48 (51%)	46 (49%)	
Respiratory pathogen documented			0.004			0.009
Yes	4 (50%)	4 (50%)		7 (37%)	12 (63%)	
No	717 (91%)	71 (9.0%)		84 (69%)	37 (31%)	
BMI			0.9			0.6
< 20	31 (91%)	3 (8.8%)		0 (0%)	1	
20–29.9	335 (89%)	40 (11%)		35 (64%)	20 (36%)	
30–34.9	114 (88%)	16 (12%)		25 (69%)	11 (31%)	
> 35	84 (91%)	8 (8.7%)		13 (65%)	7 (35%)	
Unknown	157	8		18	10	
Vaccination			0.012			0.12
No	231 (89%)	30 (11%)		31 (66%)	16 (34%)	
Yes	344 (94%)	21 (5.8%)		41 (80%)	10 (20%)	
Unknown	146	24		19	23	
Smoking			0.3			0.2
No, never	326 (93%)	23 (6.6%)		36 (75%)	12 (25%)	
No, former	187 (89%)	22 (11%)		22 (65%)	12 (35%)	
Yes, active	55 (93%)	4 (6.8%)		6	0 (0%)	
Unknown	153	26		27	25	
Clinical Frailty Scale			< 0.001			0.037
Uncomplicated	516 (92%)	42 (7.5%)		41 (80%)	10 (20%)	
Complicated	92 (86%)	15 (14%)		17 (61%)	11 (39%)	
Critical	24 (73%)	9 (27%)		17 (55%)	14 (45%)	
Unknown	89	9		16	14	
CCI			< 0.001			0.072
0–2	461 (94%)	32 (6.5%)		64 (71%)	26 (29%)	
3–4	168 (86%)	27 (14%)		20 (59%)	14 (41%)	
> 4	92 (85%)	16 (15%)		7 (44%)	9 (56%)	
Baseline WHO score			0.14			< 0.001
4	298 (89%)	38 (11%)		–	–	
5	423 (92%)	37 (8.0%)				
6–7	–	–		89 (74%)	32 (26%)	
8–9	–	–		2 (11%)	17 (89%)	

*CCI* charlson comorbidity index, *WHO* world health organization, *BMI* body mass index, *ABT* antibiotic treatment, *P-value* P-value for group differences between patients with and without ABT (Fisher exact test)

To further investigate the robustness of our results and to exclude bias, we additionally performed a propensity score analysis, which generated matched data balanced with respect to the significant covariables of Table 2. The resulting OR for ABT of 4.23 (95% CI [1.85–9.67]) resembles the OR in the multiple logistic regression. We repeated the analysis excluding patients with documented pathogens and received very similar results (OR = 4.12, 95% CI [1.88–9.05]) .

Results for patients with severe COVID-19 are shown in Table 2 and Supplementary Table S8 (Online Resource). Because of low sample size of these patients, confidence intervals for ORs in the multiple analysis are large. The characteristics of patients with constant health status during two weeks are displayed in Supplementary Tables S9 and S10 (Online Resource) alongside the two groups of patients with health decline and improvement.

### Decline in health and death during observation period

The factors ABT, higher frailty, documented pathogen and no vaccination were significantly associated with both a decline in health status and death during the observation period (hospital stay and follow-up) in the univariable analysis in the group of patients with moderate COVID-19 (Online Resource Supplementary Table S11). A higher CCI and older age had a significantly higher proportion for the outcome death only. In the multiple regression analyses, male gender had an approximately two-fold higher odds for both outcomes (Online Resource Supplementary Tables S12, S13). Age and CCI were also included in the final regression models of both outcomes, but effects on death were more pronounced than on decline in health status. Again, vaccination was a protective factor. For decline in health status, smoking and frailty were significant influence factors. Interestingly, there was no link between ABT and death. For decline in health status, however, ABT demonstrated an OR of 2.62.

Patients with severe COVID-19 disease showed similar significant influence factors for decline in health status and death in the univariable analysis (Online Resource Supplementary Table S14). ABT exhibited a strong association with both health status decline (OR = 23.96) and death (OR = 10.17) in the multiple analyses (Online Resource Supplementary Tables S15-S16).

**Fig. 3:**
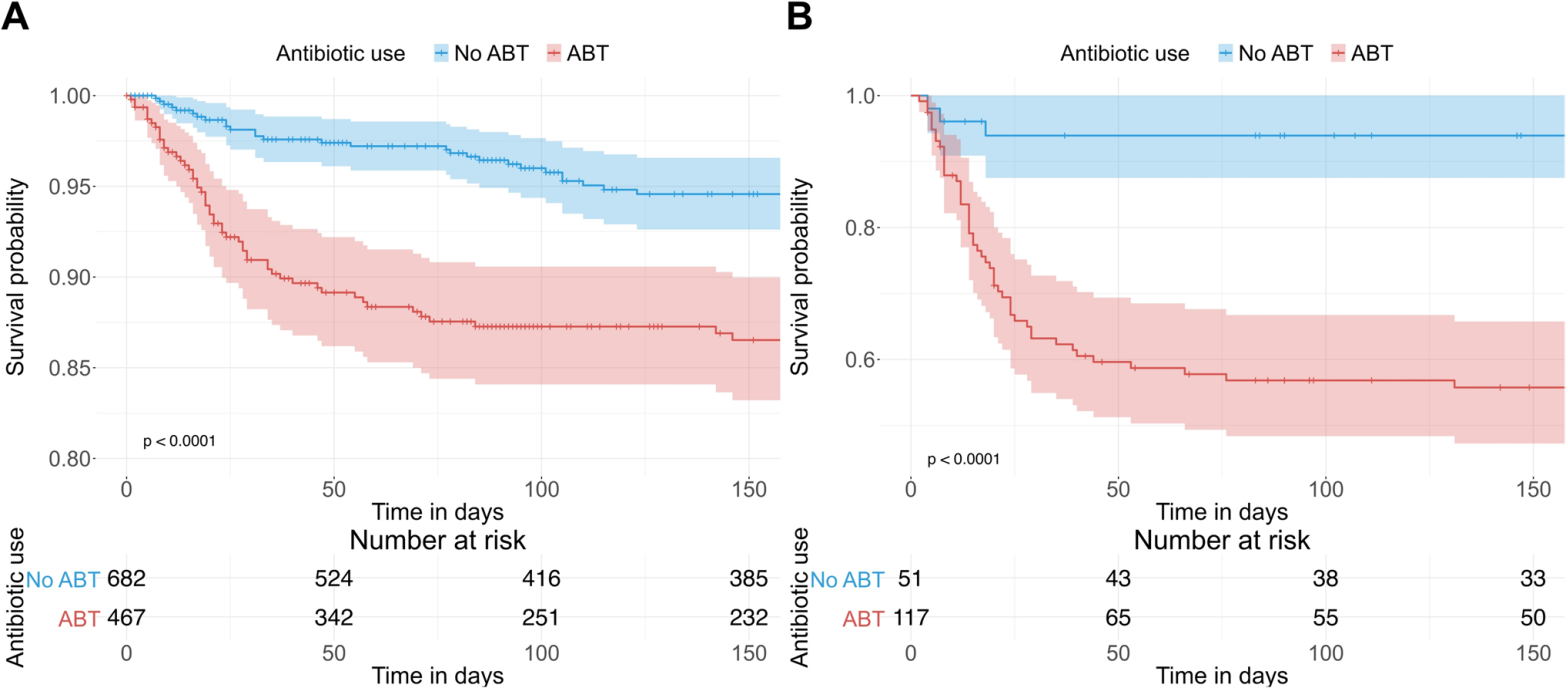
Kaplan-Meier curves for outcome death during observation period (hospital stay and follow-up). **A** Baseline WHO score 4 - 5; **B** Baseline WHO score 6-9. Shown are the survival probabilities and the 95% confidence intervals (shaded). p: P-value for differences in the distribution of survival times between patients with and without ABT (log rank test). Note that the y axis range is different in **A** and **B**

In a Kaplan–Meier analysis, the estimated survival time was significantly higher without ABT than with ABT (Fig. 3). Due to a violation of the proportional hazards assumption, a multiple Cox regression could not be performed.

### Possible reasons for decline in health in the observation period

An exploratory analysis of the data concerning development of acute renal failure (ARF) or *Clostridioides difficile*-infection (CDI) as common possible reasons for health decline was performed. 24 patients out of 467 with moderate COVID-19 developed ARF within three weeks after baseline following ABT (5.1%), whereas this occurred in only 13 of 682 patients of the non-ABT group (1.9%).

CDI was definitely documented in only 2 patients during the relevant time period, one in each group (with/without ABT). However, data availability of antibiotic substances specifically targeting *C. difficile*—vancomycin (oral), metronidazole (oral), and fidaxomycin (oral or intravenous)—was better. In the group of patients without ABT, 2 out of 682 (0.3%) of patients received such antibiotics, whereas 10 out of 467 (2.1%) of patients in the ABT group were treated with them.

### Influence of microbiological results on continuation of ABT

“No pathogen detected” was documented in a total of 66 patients in WHO group 4, 5 who were tested for relevant respiratory pathogens, considering both the predefined sampling sites and the specified time window. Of those, n = 37 (56.1%) were treated with a pneumonia-typical antibiotic whereas n = 29 (43.9%) were not. Of the 37 patients with antibiotic treatment, n = 1 had to be excluded of further evaluation due to missing data concerning end of ABT. Considering the remaining 36 patients, in n = 8 (22,2%) ABT was stopped after a negative microbiological result, in n = 28 (77.8%) ABT was continued. Median time for discontinuation of ABT was 5 days (mean 8.3 days).

## Discussion

In this study, a high proportion of patients with initial ABT commonly used for respiratory infections such as ß-lactams, macrolides, or moxifloxacin, was observed. This fact is conclusive with other studies where, despite detecting very few co-infections in hospitalised COVID-19 patients, antibiotics were widely administered [5, 12, 13]. In the subgroup of severe COVID-19, 117 out of 168 patients (70%) were treated with antibiotics and 467 out of 1149 patients (41%) in the primary study population with moderate disease. Microbiological investigations of patients with moderate disease identified only eleven patients with a superinfection with a bacterium that would plausibly cause pneumonia—eight patients treated with antibiotics and three who did not receive antibiotics. After controlling for COVID-19 risk factors including age, gender, and underlying medical conditions, our analyses found that clinical improvement after two weeks in patients with moderate disease (primary outcome) was significantly better for younger, female, and vaccinated patients. Importantly, patients given antibiotics had a five times greater risk of clinical deterioration after 14 days compared to those not treated with antibiotics. Similarly, being aged 65 or older trebled the likelihood of COVID-19 deterioration compared to those aged 18–50 years. The results for our secondary outcomes health decline and death were along the same lines and strengthen our conclusions.

Several other studies did not report any beneficial or even a potentially adverse effect of ABT in hospitalized COVID-19 patients [1, 2, 14–16]. Typically investigated outcomes were mortality or ICU admission.

Duan and colleagues investigated the effect of an early antibiotic use in a Chinese cohort of 1472 patients, hospitalized between December 2022 und March 2023 because of COVID-19. 87.4% of these patients—especially those with more risk factors and a more severe disease at admission according to WHO criteria—received an early antibiotic therapy which did neither influence significantly the overall mortality nor the need for intensive care treatment [2]. Milas et al. described similar findings in a Belgian hospital already in 2022, despite a smaller sample size and an investigated time frame in early 2020. They stated that there has been only a small amount of proven bacterial co-infections in the study cohort and more than only the critically ill patients received antibiotic therapy. This did neither lead to a shorter stay in hospital nor reduce the mortality rate [15]. Moretto and his colleagues analysed around 220 patients from Dijon, France, who where hospitalized for COVID-19 during two months in 2020. Nearly 80% of their study cohort received ABT, especially those patients with more risk factors and a more severe presentation at admission. Like in other studies, there could not be demonstrated a better outcome for patients who did receive ABT compared with those patients who did not [17]. Another French study group investigated the safety or possible harm of ABT in 150 COVID-19 patients over 80 years of age hospitalized in Amiens, France and stated that the overall mortality was higher in the ABT group [14]. In one of the largest investigated cohorts, Widere and colleagues retrospectively described the early use of ABT in hospitalized patients with COVID-19 in the United States over two years. The overall empiric antibiotic usage declined over the course of the pandemic, but early ABT remained a frequent therapeutic concept. It was shown that its use was associated with more non-favorable outcomes like increased risk for in-hospital mortality, prolonged mechanical ventilation or late *C. difficile* infection [16].

We also investigated the potential link between antibiotic treatment and adverse effects such as acute renal failure and *C. difficile* infection (CDI). Indeed, we observed an increased incidence of ARF in patients who received antibiotics. While CDI was rarely documented (only one positive test in each group (ABT and non-ABT patients) recorded between baseline and three weeks thereafter), typical antibiotics used for CDI were prescribed more often in ABT patients than in the control group without ABT, suggesting possible *C. diffile* infections.

Mehrizi and colleagues analyzed medical prescription data for hospitalized COVID-19 patients from Iran over a timespan of 26 months. Antibiotics ranked 3rd after antithrombotics and corticosteroids and the study group demonstrated that ABT was associated with a prolonged hospital stay and a higher mortality rate. Unfortunately, the registry did not provide any data regarding the disease severity, information about the clinical status, the radiological and microbiological findings or laboratory markers [18]. Last, Pinte et al. conducted a prospective multicentre study in Spain over five months in 2021 and included nearly 550 patients, divided into three risk categories (mild, moderate and severe). It should be mentioned that this categorization did not correspond to the WHO ordinal severity scale. Around 60% of the patients received ABT. Overall, ABT did not lower the mortality risk, in fact it was associated with a higher mortality when prescribed without clear evidence of a bacterial co-infection [19].

In our study, we focused on the more informative WHO score as the primary outcome. Hospitalized COVID-19 patients can exhibit considerable variability in disease severity making it challenging to assess the utility of ABT. Therefore, in contrast to other studies including all hospitalized patients, our primary study population consisted of patients with moderate disease in whom use of ABT is most controversial. While early antibiotic therapy is crucial in suspected bacterial infection, it is equally important to regularly review its indication and discontinue its use when there is no evidence for such an infection. In our study, ABT was not discontinued upon receiving a negative microbiological result. Since the median time between date of microbiological result and end of ABT was 5 days (mean 8.3 days), it appears unlikely that therapy discontinuation was directly related to receiving a negative result, but rather influenced by other factors (e.g. clinical stabilization of the patient).

Despite the strengths of our study, its observational nature represents a key limitation. Although relevant risk factors for COVID-19 progression were adjusted for in the multiple regression analyses, other important contributory factors and confounders might have been omitted. Especially, the low number of patients with documented respiratory bacterial superinfection might be due to missing microbiological diagnostic samples or missing documentation of results. Moreover, only patients hospitalized in Germany were included whose results might not be generalisable to other populations.

The increased risk for deterioration under ABT might partly be due to unknown additional confounders resulting in worse outcomes and being associated with ABT such as undocumented bacterial superinfection. Importantly, no positive effect of ABT could be detected and clinical deterioration might develop from adverse effects of unnecessarily prescribed antibiotics. The COVID-19 pandemic is ripe with lessons for future viral pandemics. The overuse of antibiotics seen in the pandemic without a beneficial impact on outcomes highlights the need for more rational antibiotic use and points to strengthening antibiotic stewardship programmes. Early initiation of antibiotic therapy upon suspicion is indicated especially in severe, frail patients and those at risk of deterioration. Instead, rational antibiotic use should be limited to patients with likely bacterial coinfection only and started after performance of microbiological diagnostics for confirmation of a bacterial infection (e.g. blood and sputum cultures in case of suspected pneumonia). De-escalation, which may involve narrowing the antibiotic spectrum or even completely discontinuing treatment, is advisable when the microbiological results do not support the presence of an infection [20]. Considering the results of our study, antibiotics should be discontinued in COVID-19 patients with WHO score 4-5 once a co-infection has been deemed unlikely.

## Supplementary Information

Below is the link to the electronic supplementary material.

Supplementary file1 (PDF 340 KB)

## Data Availability

All data supporting the findings of this study are available within the paper and its Supplementary Information.
