# Supplementary material for: The effect of antibiotic therapy on clinical outcome in patients hospitalized with moderate COVID-19 disease: a prospective multi-center cohort study
Source: Infection. 2025 Jun 26;53(6):2543–55. doi: 10.1007/s15010-025-02590-0 (PMC12675722; doi:10.1007/s15010-025-02590-0)
Supplement: Supplementary file 1 — Supplementary file1 (PDF 340 KB) [file 15010_2025_2590_MOESM1_ESM.pdf]

Supplementary Tables

Supplementary Table S1 WHO Clinical Progression Scale [7]

Patient State	Descriptor	Score
Uninfected	Uninfected; no viral RNA detected	0
Ambulatory mild disease	Asymptomatic; viral RNA detected	1
	Symptomatic; independent	2
	Symptomatic; assistance needed	3
Hospitalized: moderate disease	Hospitalized; no oxygen therapy	4
	Hospitalized; oxygen by mask or nasal prongs	5
Hospitalized: severe diseases	Hospitalized; oxygen by NIV or high flow	6
	Intubation and mechanical ventilation, pO_2/FiO_2 ≥150 or SpO_2/FiO_2 ≥200	7
	Mechanical ventilation pO_2/FiO_2 <150 (SpO_2/FiO_2 <200) or vasopressors	8
	Mechanical ventilation pO_2/FiO_2 <150 and vasopressors, dialysis, or ECMO	9
Dead	Dead	10

Supplementary Table S2: Pneumonia antibiotics administered

Antibiotic groups	Antibiotic	Number of cycles	Number of patients
**Acylureidopenicillin and BLI**	Piperacillin and BLI	**328 (33.03%)**	**236 (29.26%)**
**Aminopenicillin**	Ampicillin	**32 (3.22%)**	**30 (3.68%)**
**Aminopenicillin and BLI**	Amoxicillin and BLI	28 (2.82%)	28 (3.43%)
	Ampicillin and BLI	233 (23.46%)	192 (23.53%)
	**Total**	**261 (26.28%)**	**220 (26.96%)**
**Carbapenem**	Imipenem and Cilastatin	2 (0.20%)	1 (0.12%)
	Meropenem	47 (4.73%)	43 (5.27%)
	**Total**	**50 (4.75%)**	**44 (5.39%)**
**2nd generation cephalosporin**	Cefuroxime	**12 (1.12%)**	**11 (1.35%)**
**3rd generation cephalosporin**	Cefotaxime	42 (4.23%)	25 (3.06%)
	Ceftriaxone	52 (5.24%)	50 (6.13%)
	**Total**	**94 (9.47%)**	**75 (9.19%)**
**3rd generation cephalosporin (subgroup 3b)**	Ceftazidime	**9 (0.91%)**	**7 (0.86%)**
**Fluoroquinolone**	Levofloxacin	14 (1.41%)	12 (1.47%)
	Moxifloxacin	10 (1.01%)	10 (1.23%)
	**Total**	**24 (2.42%)**	**22 (2.7%)**
**Macrolide**	Azithromycin	96 (9.67%)	95 (11.64%)
	Clarithromycin	85 (8.56%)	73 (8.95%)
	**Total**	**184 (18.23%)**	**168 (20.59%)**
**Tetracycline**	Doxycycline	**3 (0.30%)**	**3 (0.37%)**
**Overall**		**997**	**816**

Time period for ABT: seven days before baseline to four days after baseline. Patients were sometimes treated with multiple antibiotics, therefore the number of patients with ABT was only 584. An antibiotic cycle refers to the administration of an antibiotic to a patient at least once, which may or may not extend over several days. Regardless of the duration, each cycle is counted as one in the tally of antibiotic cycles.

Supplementary Table S3

Distributional characteristics of covariables in the unmatched (All Data) and matched data

Summary of Balance for All Data:

	**Prop. with ABT**	**Prop. without ABT**	**Std. Mean Diff.**
PS*	0.4665	0.4275	0.3494
gender: female	0.3902	0.4300	-0.0814
gender: male	0.6098	0.5700	0.0814
age: 18 – 49.9	0.2642	0.3616	-0.2208
age: >80	0.1301	0.0489	0.2414
age: 50 – 64.9	0.3455	0.3876	-0.0885
age: 65 – 79.9	0.2602	0.2020	0.1327
superinfection: no bac. detected	0.9837	0.9935	-0.0771
superinfection: bac. detected	0.0163	0.0065	0.0771
not vaccinated	0.4431	0.4365	0.0133
vaccinated	0.5569	0.5635	-0.0133
CFS: uncomplicated	0.7520	0.8176	-0.1518
CFS: complicated	0.1789	0.1466	0.0842
CFS: critical	0.0691	0.0358	0.1312
CCI: 0-2	0.5732	0.6384	-0.1320
CCI: >4	0.1504	0.1173	0.0927
CCI: 3-4	0.2764	0.2443	0.0718

Summary of Balance for Matched Data:

	Prop. with ABT	Prop. without ABT	Std. Mean Diff.
PS*	0.4397	0.4391	0.0059
gender: female	0.4131	0.4272	-0.0289
gender: male	0.5869	0.5728	0.0289
age: 18 – 49.9	0.2911	0.2676	0.0532
age: >80	0.0563	0.0610	-0.0140
age: 50 – 64.9	0.3991	0.4507	-0.1086
age: 65 – 79.9	0.2535	0.2207	0.0749
superinfection: no bac. detected	0.9953	0.9953	0.0000
superinfection: bac. detected	0.0047	0.0047	0.0000
not vaccinated	0.4507	0.4460	0.0095
vaccinated	0.5493	0.5540	-0.0095
CFS: uncomplicated	0.8216	0.7840	0.0870
CFS: complicated	0.1362	0.1737	-0.0980
CFS: critical	0.0423	0.0423	0.0000
CCI: 0-2	0.5915	0.5962	-0.0095
CCI: >4	0.1315	0.1127	0.0525
CCI:3-4	0.2770	0.2911	-0.0315

Sample Sizes:

	Patients without ABT	Patients with ABT
All	307	246
Matched	213	213
Unmatched	94	33

Output adapted from the summary.matchit function of the R package MatchIt.

PS: propensity score, CCI: Charlson Comorbidity Index, CFS: Clinical Frailty Scale, ABT: antibiotic treatment Prop. with/without ABT: proportion of patients with/without ABT in the respective category, Std. Mean Diff.: standardized mean difference, * Because the PS is a continuous variable, the mean instead of the proportion is given.

Supplementary Table S4: Documented pneumonia-relevant pathogens

Pathogen	Frequency	Sample material
Enterobacter cloacae	4 (4.76%)	BAL (1), tracheal secretion (3)
Escherichia coli	1 (1.19%)	Tracheal secretion (1)
Hafnia alvei	1 (1.19%)	BAL (1)
Klebsiella	2 (2.38%)	BAL (1), tracheal secretion (1)
Klebsiella oxytoca	1 (1.19%)	BAL (1)
Legionella	1 (1.19%)	Urine (1)
Legionella pneumophila	1 (1.19%)	Urine (1)
Moraxella	2 (2.38%)	Blood culture (1), tracheal secretion (1)
Morganella	1 (1.19%)	tracheal secretion (1)
Proteus mirabilis	2 (2.38%)	Blood culture (1), tracheal secretion (1)
Pseudomonas aeruginosa	2 (2.38%)	tracheal secretion (2)
Serratia species	2 (2.38%)	tracheal secretion (2)
Staphylococcus aureus	18 (21.43%)	BAL (1), blood culture (7), tracheal secretion (10)
Stenotrophomonas	2 (2.38%)	Blood culture (1), tracheal secretion (1)
Streptococcus pneumoniae	5 (5.95%)	Blood culture (1), tracheal secretion (1), urine (3)
**Overall**	45	

BAL: Bronchoalveolar lavage. Time period for extraction of material for pathogen test: seven days before baseline to four days after baseline. Multiple pathogens were detected for some patients, therefore the number of patients with detected pathogens was only 34.

Supplementary Table S5: Multiple logistic regression analysis: Factors associated with ABT at baseline (moderate COVID-19)

Variable	Category	Odds Ratio (95% CI)	P-value
**Intercept**		0.298 (0.21 - 0.43)	<0.001
**Vaccination**	No (ref)	1	
	Yes	0.63 (0.46 - 0.85)	0.002
**Gender**	Female (ref)	1	
	Male	1.44 (1.09 - 1.92)	0.011
**Age**	18 - 49.9 years (ref)	1	
	50 - 64.9 years	1.69 (1.19 - 2.41)	0.004
	65 - 79.9 years	2.47 (1.66 - 3.67)	<0.001
	⩾ 80 years	3.60 (2.16 - 6.06)	<0.001
**CCI**	0 - 2 (ref)	1	
	3 - 4	1.64 (1.17 - 2.29)	0.004
	> 4	1.39 (0.91 - 2.11)	0.125
**Baseline WHO score**	4 (ref)	1	
	5	1.49 (1.12 - 1.99)	0.006

Nagelkerke's R^2 = 0.415

CCI: Charlson Comorbidity Index, WHO: World Health Organization, ref: Reference category for the variable in the multiple logistic regression model, 95% CI: 95% confidence interval

Variables in the full model but excluded during model selection: body mass index, smoking, Clinical Frailty Scale

Supplementary Table S6: Multiple logistic regression analysis: Factors associated with ABT at baseline (severe COVID-19)

Variable	Category	Odds Ratio (95% CI)	P-value
**Intercept**		1.07 (0.69 - 1.65)	0.78
**CCI**	0 - 2 (ref)	1	
	3 – 4	3.21 (1.33 - 8.69)	0.014
	> 4	6.58 (1.72 - 43.37)	0.016
**Baseline WHO**	6 - 7 (ref)	1	
**score**	8 – 9	5.52 (2.16 - 17.14)	0.001

Nagelkerke's R^2 = 0.206

CCI: Charlson Comorbidity Index, WHO: World Health Organization, ref: Reference category for the variable in the multiple logistic regression model, 95% CI: 95% confidence interval

Variables in the full model but excluded during model selection: gender, age, body mass index, smoking, Clinical Frailty Scale, vaccination

Supplementary Table S7: Multiple logistic regression analysis: Factors associated with improvement vs. deterioration after 14 days (moderate COVID-19)

Variable	Category	Odds Ratio (95% CI)	p-value
**Intercept**		0.02 (0.01 – 0.04)	< 0.001
**ABT**	No (ref)	1	
	Yes	5.00 (2.50 – 10.93)	< 0.001
**Gender**	Female (ref)	1	
	Male	2.12 (1.11 – 4.26)	0.028
**Age**	18 – 49.9 years (ref)	1	
	50 - 64.9 years	1.43 (0.57 – 3.71)	0.443
	65 - 79.9 years	3.76 (1.62 – 9.36)	0.003
	⩾ 80 years	4.85 (1.72 – 13.97)	0.003
**Vaccination**	No (ref)	1	
	Yes	0.33 (0.17 – 0.62)	0.001

Nagelkerke's R^2 = 0.499

ABT: Antibiotic treatment, ref: Reference category for the variable in the multiple logistic regression model, 95% CI: 95% confidence interval

Variables in the full model but excluded during model selection: body mass index, smoking, Clinical Frailty Scale, baseline WHO (World Health Organization) score, Charlson Comorbidity index

Supplementary Table S8: Multiple logistic regression analysis: Factors associated with improvement vs. deterioration after 14 days (severe COVID-19)

Variable	Category	Odds Ratio (95% CI)	p-value
**Intercept**		0.06 (0.01 - 0.17)	< 0.001
**ABT**	No (ref)	1	
	Yes	10.81 (3.42 - 49.17)	< 0.001
**Baseline WHO Score**	6 – 7 (ref)	1	
	8 - 9	17.29 (4.29 - 120.59)	< 0.001

Nagelkerke's R^2 = 0.405

ABT: Antibiotic treatment, ref: Reference category for the variable in the multiple logistic regression model, 95% CI: 95% confidence interval

Variables in the full model but excluded during model selection: gender, age, body mass index, smoking, Clinical Frailty Scale, vaccination, Charlson Comorbidity index

Supplementary Table S9: Change of WHO score after 14 days (moderate COVID-19)

	Overall, N = 1,149	Improvement N = 721	No change N = 353	Deterioration N = 75	P-value
**Gender**					0.028
Female	453	295 (65%)	139 (31%)	19 (4.2%)	
Male	696	426 (61%)	214 (31%)	56 (8.0%)	
**Age group**					<0.001
18 - 49.9	384	236 (61%)	138 (36%)	10 (2.6%)	
50 - 64.9	380	259 (68%)	100 (26%)	21 (5.5%)	
65 - 79.9	269	168 (62%)	73 (27%)	28 (10%)	
⩾80	116	58 (50%)	42 (36%)	16 (14%)	
**ABT at baseline**					<0.001
No	682	429 (63%)	239 (35%)	14 (2.1%)	
Yes	467	292 (63%)	114 (24%)	61 (13%)	
**Respiratory pathogen documented**					0.006
Yes	11	4 (36%)	3 (27%)	4 (36%)	
No	1,138	717 (63%)	350 (31%)	71 (6.2%)	
**BMI**					0.002
<20	52	31 (60%)	18 (35%)	3 (5.8%)	
20-29.9	583	335 (57%)	208 (36%)	40 (6.9%)	
30-34.9	167	114 (68%)	37 (22%)	16 (9.6%)	
>35	116	84 (72%)	24 (21%)	8 (6.9%)	
Unknown	231	157	66	8	
**Vaccination**					<0.001
No	318	231 (73%)	57 (18%)	30 (9.4%)	
Yes	614	344 (56%)	249 (41%)	21 (3.4%)	

Unknown	217	146	47	24	
**Smoking**					0.3
No, never	509	326 (64%)	160 (31%)	23 (4.5%)	
No, former	304	187 (62%)	95 (31%)	22 (7.2%)	
Yes, active	97	55 (57%)	38 (39%)	4 (4.1%)	
Unknown	239	153	60	26	
**Clinical Frailty Scale**					<0.001
Uncomplicated	798	516 (65%)	240 (30%)	42 (5.3%)	
Complicated	137	92 (67%)	30 (22%)	15 (11%)	
Critical	49	24 (49%)	16 (33%)	9 (18%)	
Unknown	165	89	67	9	
**CCI**					0.005
0-2	713	461 (65%)	220 (31%)	32 (4.5%)	
3-4	273	168 (62%)	78 (29%)	27 (9.9%)	
>4	163	92 (56%)	55 (34%)	16 (9.8%)	
**Baseline WHO score**					<0.001
4	632	298 (47%)	296 (47%)	38 (6.0%)	
5	517	423 (82%)	57 (11%)	37 (7.2%)	

CCI: Charlson Comorbidity Index, WHO: World Health Organization, BMI: Body mass index, ABT: Antibiotic treatment, P-value: P-value for group differences between patients with and without ABT (Fisher exact test)

Supplementary Table S10: Change of WHO score after 14 days (severe COVID-19)

	Overall N = 168	Improvement N = 91	No change N = 28	Deterioration N = 49	P-value
**Gender**					0.2
Female	49	32 (65%)	7 (14%)	10 (20%)	
Male	119	59 (50%)	21 (18%)	39 (33%)	
**Age group**					0.9
18 - 49.9	44	27 (61%)	7 (16%)	10 (23%)	
50 - 64.9	67	35 (52%)	11 (16%)	21 (31%)	
65 - 79.9	43	23 (53%)	8 (19%)	12 (28%)	
⩾80	14	6 (43%)	2 (14%)	6 (43%)	
**ABT at baseline**					<0.001
No	51	43 (84%)	5 (9.8%)	3 (5.9%)	
Yes	117	48 (41%)	23 (20%)	46 (39%)	
**Respiratory pathogen documented**					0.018
Yes	23	7 (30%)	4 (17%)	12 (52%)	
No	145	84 (58%)	24 (17%)	37 (26%)	
**BMI**					0.12
<20	2	0 (0%)	1 (50%)	1 (50%)	
20-29.9	65	35 (54%)	10 (15%)	20 (31%)	
30-34.9	38	25 (66%)	2 (5.3%)	11 (29%)	
>35	27	13 (48%)	7 (26%)	7 (26%)	
Unknown	36	18	8	10	
**Vaccination**					0.2
No	53	31 (58%)	6 (11%)	16 (30%)	
Yes	63	41 (65%)	12 (19%)	10 (16%)	

Unknown	52	19	10	23	
**Smoking**					0.004
No, never	52	36 (69%)	4 (7.7%)	12 (23%)	
No, former	38	22 (58%)	4 (11%)	12 (32%)	
Yes, active	12	6 (50%)	6 (50%)	0 (0%)	
Unknown	66	27	14	25	
**Clinical Frailty Scale**					0.031
Uncomplicated	55	41 (75%)	4 (7.3%)	10 (18%)	
Complicated	33	17 (52%)	5 (15%)	11 (33%)	
Critical	39	17 (44%)	8 (21%)	14 (36%)	
Unknown	41	16	11	14	
**CCI**					0.2
0-2	107	64 (60%)	17 (16%)	26 (24%)	
3-4	41	20 (49%)	7 (17%)	14 (34%)	
>4	20	7 (35%)	4 (20%)	9 (45%)	
**Baseline WHO score**					<0.001
6 - 7	136	89 (65%)	15 (11%)	32 (24%)	
8 - 9	32	2 (6.3%)	13 (41%)	17 (53%)	

CCI: Charlson Comorbidity Index, WHO: World Health Organization, BMI: Body mass index, ABT: Antibiotic treatment, P-value: P-value for group differences between patients with and without ABT (Fisher exact test)

Supplementary Table S11: Health decline and death during observation period (moderate COVID-19)

	Overall<br>N = 1,149	Health decline<br>N = 194	P-value<br>health decline	Death<br>N = 94	P-value<br>death
**Gender**			<0.001		0.028
Female	453	53 (12%)		27 (6.0%)	
Male	696	141 (20%)		67 (9.6%)	
**Age group**			<0.001		<0.001
18 - 49.9	384	30 (7.8%)		5 (1.3%)	
50 - 64.9	380	59 (16%)		24 (6.3%)	
65 - 79.9	269	69 (26%)		39 (14%)	
⩾80	116	36 (31%)		26 (22%)	
**ABT at baseline**			<0.001		<0.001
No	682	75 (11%)		32 (4.7%)	
Yes	467	119 (25%)		62 (13%)	
**Respiratory pathogen documented**			0.005		0.009
Yes	11	6 (55%)		90 (7.9%)	
No	1,138	188 (17%)		4 (36%)	
**BMI**			0.13		0.12
<20	52	8 (15%)		6 (12%)	
20-29.9	583	99 (17%)		44 (7.5%)	
30-34.9	167	39 (23%)		19 (11%)	
>35	116	15 (13%)		5 (4.3%)	
Unknown	231	33		20	
**Vaccination**			0.2		0.2
Not vaccinated	318	51 (16%)		28 (8.8%)	
Vaccinated	614	80 (13%)		41 (6.7%)	
Unknown	217	63		25	
**Smoking**			<0.001		0.084

No, never	509	66 (13%)		30 (5.9%)	
No, former	304	63 (21%)		29 (9.5%)	
Yes, active	97	5 (5.2%)		4 (4.1%)	
Unknown	239	60		31	
**Clinical Frailty Scale**			<0.001		<0.001
Uncomplicated	798	103 (13%)		42 (5.3%)	
Complicated	137	46 (34%)		26 (19%)	
Critical	49	15 (31%)		7 (14%)	
Unknown	165	30		19	
**CCI**			<0.001		<0.001
0-2	713	88 (12%)		26 (3.6%)	
3-4	273	57 (21%)		38 (14%)	
>4	163	49 (30%)		30 (18%)	
**Baseline WHO score**			0.5		0.3
4	632	111 (18%)		47 (7.4%)	
5	517	83 (16%)		47 (9.1%)	

CCI: Charlson Comorbidity Index, WHO: World Health Organization, BMI: Body mass index, ABT: Antibiotic treatment, P-value: P-value for group differences between patients with and without ABT (Fisher exact test). Health decline is defined as a higher WHO score during observation period (hospital stay and follow-up) than baseline.

Supplementary Table S12: Multiple logistic regression analysis: Factors associated with health decline during observation period (moderate COVID-19)

Variable	Category	Odds Ratio (95% CI)	P-value
**Intercept**		0.04 (0.02 - 0.08)	<0.001
**CCI**	0 – 2 (ref)	1	
	3 - 4	0.93 (0.52 - 1.65)	0.816
	> 4	2.01 (1.09 - 3.64)	0.022
**ABT**	No (ref)	1	
	Yes	2.62 (1.63 - 4.25)	<0.001
**Gender**	Female (ref)	1	
	Male	2.11 (1.26 - 3.65)	0.006
**Age**	18 – 49.9 years (ref)	1	
	50 - 64.9 years	1.13 (0.59 - 2.21)	0.712
	65 - 79.9 years	2.58 (1.34 - 5.09)	0.005
	⩾80 years	1.69 (0.71 - 3.97)	0.228
**Vaccination**	No (ref)		
	Yes	0.56 (0.34 - 0.91)	0.019
**Smoking**	No, never (ref)	1	
	No, former	1.51 (0.93 - 2.47)	0.097
	Yes, active	0.28 (0.07 - 0.82)	0.042
**Clinical Frailty Scale**	Uncomplicated	1	
	Complicated	2.31 (1.31 - 4.01)	0.003
	Critical	1.18 (0.41 - 3.03)	0.749

Nagelkerke's R^2 = 0.694

CCI: Charlson Comorbidity Index, ABT: Antibiotic treatment, ref: Reference category for the variable in the multiple logistic regression model, 95% CI: 95% confidence interval

Variables in the full model but excluded during model selection: body mass index, baseline WHO (World Health Organization) score. Health decline is defined as a higher WHO score during observation period (hospital stay and follow-up) than baseline.

Supplementary Table S13: Multiple logistic regression analysis: Factors associated with death during hospital stay (moderate COVID-19)

Variable	Category	Odds Ratio (95% CI)	p-Value
**Intercept**		0.01 (0.00 - 0.02)	<0.001
**CCI**	0 – 2 (ref)	1	
	3 - 4	2.46 (1.30 – 4.72)	0.006
	> 4	3.89 (1.98 – 7.70)	<0.001
**Gender**	Female (ref)	1	
	Male	2.13 (1.21 – 3.89)	0.011
**Age**	18 – 49.9 (ref)	1	
	50 - 64.9 years	3.75 (1.34 – 13.32)	0.020
	65 - 79.9 years	9.35 (3.49 – 32.61)	<0.001
	⩾80 years	16.20 (5.59 – 59.10)	<0.001
**Vaccination**	No (ref)	1	
	Yes	0.48 (0.28 – 0.83)	0.009

Nagelkerke's R^2 = 0.455

CCI: Charlson Comorbidity Index, ref: Reference category for the variable in the multiple logistic regression model, 95% CI: 95% confidence interval

Variables in the full model but excluded during model selection: body mass index, baseline WHO (World Health Organization) score, smoking, Clinical Frailty Scale, ABT.

Supplementary Table S14: Health decline and death during observation period (severe COVID-19)

	Overall<br>N = 168	Health decline<br>N = 75	P-value<br>health decline	Death<br>N = 53	P-value death
**Gender**			0.4		0.3
Female	49	19 (39%)		12 (24%)	
Male	119	56 (47%)		41 (34%)	
**Age group**			0.13		<0.001
18 - 49.9	44	15 (34%)		6 (14%)	
50 - 64.9	67	28 (42%)		19 (28%)	
65 - 79.9	43	23 (53%)		19 (44%)	
⩾80	14	9 (64%)		9 (64%)	
**ABT at baseline**			<0.001		<0.001
No	51	7 (14%)		3 (5.9%)	
Yes	117	68 (58%)		50 (43%)	
**Pathogen documented**			0.003		0.030
Yes	23	17 (74%)		12 (52%)	
No	145	58 (40%)		41 (28%)	
**BMI**			0.3		0.7
<20	2	1 (50%)		1 (50%)	
20-29.9	65	31 (48%)		20 (31%)	
30-34.9	38	12 (32%)		9 (24%)	
>35	27	14 (52%)		8 (30%)	
Unknown	36	17		15	
**Vaccination**			0.033		0.008
No	53	25 (47%)		18 (34%)	
Yes	63	17 (27%)		8 (13%)	
Unknown	52	33		27	
**Smoking**			0.5		0.2

No, never	52	16 (31%)		9 (17%)	
No, former	38	16 (42%)		12 (32%)	
Yes, actively	12	3 (25%)		1 (8.3%)	
Unknown	66	40		31	
**Clinical Frailty Scale**			0.002		0.001
Uncomplicated	55	12 (22%)		6 (11%)	
Complicated	33	18 (55%)		11 (33%)	
Critical	39	20 (51%)		17 (44%)	
Unknown	41	25		19	
**CCI**			0.057		0.010
0-2	107	41 (38%)		26 (24%)	
3-4	41	21 (51%)		16 (39%)	
>4	20	13 (65%)		11 (55%)	
**Baseline WHO score**			0.2		0.5
6 - 7	121	50 (41%)		36 (30%)	
8 - 9	47	25 (53%)		17 (36%)	

CCI: Charlson Comorbidity Index, WHO: World Health Organization, BMI: Body mass index, ABT: Antibiotic treatment, P-value: P-value for group differences between patients with and without ABT (Fisher exact test). Health decline is defined as a higher WHO score during observation period (hospital stay and follow-up) than baseline.

Supplementary Table S15: Multiple logistic regression analysis: Factors associated with health decline during observation period (severe COVID-19)

Variable	Category	Odds Ratio (95% CI)	P-value
**Intercept**		0.03 (0.00 - 0.10)	<0.001
**ABT**	No (ref)	1	
	Yes	23.96 (6.61 - 154.74)	<0.001
**Clinical Frailty Scale**	Uncomplicated (ref)	1	
	Complicated	3.52 (1.23 - 10.67)	0.022
	Critical	2.84 (1.05 - 8.02)	0.043

Nagelkerke's R^2 = 0.226

ABT: Antibiotic treatment, ref: Reference category for the variable in the multiple logistic regression model, 95% CI: 95% confidence interval

Variables in the full model but excluded during model selection: gender, age, body mass index, baseline WHO (World Health Organization) score, smoking, Charlson Comorbidity index, vaccination. Health decline is defined as a higher WHO score during observation period (hospital stay and follow-up) than baseline.

Supplementary Table S16: Multiple logistic regression analysis: Factors associated with death during observation period (severe COVID-19)

Variable	Category	Odds Ratio (95% CI)	P-value
**Intercept**		0.03 (0.01 - 0.10)	<0.001
**ABT**	No (ref)	1	
	Yes	10.17 (3.38 - 44.16)	<0.001
**Age**	18 - 49.9 years (ref)	1	
	50 - 64.9 years	2.30 (0.83 - 7.12)	0.123
	65 - 79.9 years	3.86 (1.34 - 12.42)	0.016
	⩾80 years	9.00 (2.17 - 43.56)	0.004

Nagelkerke's R^2 = 0.208

ABT: Antibiotic treatment, ref: Reference category for the variable in the multiple logistic regression model, 95% CI: 95% confidence interval

Variables in the full model but excluded during model selection: gender, body mass index, baseline WHO (World Health Organization) score, smoking, Clinical Frailty Scale, Charlson Comorbidity index, vaccination.
